# Molecular mechanisms of hormones implicated in migraine and the translational implication for transgender patients

**DOI:** 10.3389/fpain.2023.1117842

**Published:** 2023-09-19

**Authors:** Cameron I. Martinez, Erika Liktor-Busa, Tally M. Largent-Milnes

**Affiliations:** Department of Pharmacology, College of Medicine, University of Arizona, Tucson, AZ, United States

**Keywords:** migraine, transgender, cortical spreading depression, central sensitization, gender-affirming healthcare, chronic pain, estrogen, testosterone

## Abstract

Migraine is a primary headache disorder recognized by the World Health Organization as one of the most poorly understood and debilitating neurological conditions impacting global disability. Chronic pain disorders are more frequently diagnosed among cisgender women than men, suggesting that female sex hormones could be responsible for mediating chronic pain, including migraine and/or that androgens can be protective. This review discusses the major gonadal hormones, estrogens, progesterone, and testosterone in the context of molecular mechanisms by which they play a role in migraine pathophysiology. In addition, the literature to date describing roles of minor sex hormones including prolactin, luteinizing hormone, follicular stimulating hormone, and gonadotropin releasing hormone in migraine are presented. Because transgender and gender non-conforming (trans*) individuals are an underserved patient population in which gender-affirming sex hormone replacement therapy (HRT) is often medically necessary to align biological sex with gender identity, results from cisgender patient populations are discussed in the context of these major and minor sex hormones on migraine incidence and management in trans* patients.

## 1. Introduction

### 1.1. Migraine

Migraine is a life-long headache disorder that causes severe, throbbing unilateral face pain, photophobia, phonophobia, nausea, and other distressing symptoms that significantly reduce patient quality of life. Migraine headaches are diagnosed based upon attack frequency and aura. When headache symptoms are present for at least three months, episodic migraine is defined as 0–14 headache days per month while chronic migraine requires 15 or more headache days (1). Aura in migraine refers to any sensory disturbances that occur before/with headache pain and is mostly attributed to symptoms that disrupt visual perception, such as scotomas or blurred vision on one side of the visual field (2). The physiological correlate of migraine aura is cortical spreading depression (CSD) (3). Migraine is also considered evolutive in nature since each headache attack is categorized by phases (i.e., premonitory/aura, ictal headache, and postdrome) which may transform from episodic to chronic within a given individual's lifetime (4). Considering the significance of these factors on patient wellbeing, the World Health Organization recognizes migraine as the “most prevalent, disabling, long-term neurological condition” that impacts an estimated 15% of the global population (4).

### 1.2. Molecular mechanisms associated with migriane

Cortical spreading depression is defined as the spreading of a slowly propagating wave of cellular depolarization and inhibition within neuronal and glial cells across the cerebral cortex. A large change in the concentration gradient between extracellular potassium and intracellular sodium/calcium ions produces a depression of electrocortical signals that travels anteriorly at a rate of 3–6 mm/min (5, 6). In addition to aura and disturbances in sensory processing, CSD propagation facilitates the release of glutamate, which is the neurotransmitter responsible for altering cortical excitability and synaptic plasticity within the central nervous system (5, 6). CSD has been proposed to create the foundational landscape for the migraine mechanism known as central sensitization. Described by Wang et al. (7), central sensitization is a primary neurological mechanism underlying chronic headache disorders in which heightened cortical excitability within trigeminal nociceptive pathways lowers pain thresholds and promotes repetitive headache neurotransmission, leading to permanent modifications in pain perception (7). The synaptic efficiency of trigeminal neurons is established through long-term potentiation, which uses molecules that proliferate dendritic growth to strengthen associations between neuronal activity and headache pathology; one of these molecules, brain-derived neurotropic factor (BDNF), is released by a neurogenic inflammatory neuropeptide called calcitonin gene-related peptide (CGRP).

Preliminary research on the possible involvement of CGRP in migraine found that patients experienced elevated CGRP levels during the interictal phase when headache symptoms were absent (8); furthermore, CGRP antagonists have shown significant therapeutic efficacy in reducing headache frequency by as much as 50% within three months (9). CGRP activates a neurogenic inflammatory response that coincides with headache, mechanical/thermal hyperalgesia, and spontaneous facial pain (10). The central nervous system contains CGRP receptors on neurons and glial cells within the trigeminal ganglion, spinal trigeminal nucleus, thalamus, hypothalamus, spinal cord, and cerebellum as well as within the cardiovascular system (11). The synthesis and primary release of CGRP occurs with activation of transient receptor potential (TRP) channels. TRP channels are mainly expressed in a subset of primary sensory neurons within the trigeminal ganglion to promote nociception through their function as thermoreceptors and chemoreceptors; specifically, activation of TRP vanilloid 1 (TRPV1) and TRP ankyrin (TRPA1) promote the development of allodynia and hyperalgesia through neurogenic inflammation (12). Since ethanol is commonly reported as a migraine trigger, one report showed that ethanol promoted CGRP release and lowered the threshold temperature necessary to activate TRP channels, thereby triggering a CGRP-dependent meningeal vasodilatory response (12). By stimulating central projections within the trigeminal ganglion, CGRP activates neurons within the trigeminal nucleus to upregulate the production of pronociceptive substances. These inflammatory molecules are transported along central terminals into the spinal trigeminal nucleus where CGRP receptors increase glutamate and BDNF levels to reinforce migraine headache episodes (11). In addition to synaptic plasticity, BDNF also increases CGRP expression, resulting in a positive feedback loop that maintains the persistent stimulation of trigeminal neurons (13).

Given that trigeminovascular neurons have intrinsic projections to the cerebrovascular system, cortical hyperexcitability induced within central neurons activates and amplifies the underlying vascular processes that sustain migraine headache. The current neurovascular understanding of migraine suggests that neurological processes are necessary to alter trigeminal activity (14). Clinical models have often used nitric oxide donor drugs such as nitroglycerine to induce migraine headache because of their efficiency in producing heightened pain states with allodynia, thus implicating vascular processes in headache (12). Harold Wolff and colleagues were the first to refine and test the vascular theory of migraine which they defined in two components: intracranial vasospasm of the cerebral arteries generates migraine aura, and extracranial vasodilation—along with lowered trigeminal pain thresholds and neurogenic inflammation—initiates migraine headache (15). While the first element was later disproven in favor of CSD propagation theory, the second component reflects the present understanding of vascular migraine mechanisms. Additional studies have expanded research on the intersecting molecular pathways between vasodilation, trigeminal cortical hyperexcitability, and neurogenic inflammation to propose that migraineurs suffer from increased arterial stiffness and endothelial dysfunction as a result of oxidative stress (16, 17). Endothelial dysfunction is defined as “the inability of the artery to sufficiently dilate in response to an appropriate endothelial stimulus,” which includes an aberrant response to the disproportionate upregulation of proinflammatory and procoagulant molecules released in migraine (16). When CGRP receptors on endothelial cells within cranial and coronary arteries are activated, the endothelial protein kinase A/nitric oxide synthase signaling pathway is stimulated to increase nitric oxide (18). Uzar et al. (19) supported this with evidence that migraine patients demonstrate elevated levels of nitric oxide during the ictal phase. Because nitric oxide is a significant vasodilator, excessive vasodilation within cranial arteries reinforces neurogenic inflammation and contributes to the throbbing and pulsing qualities of migraine headache (20). However, because of this heightened endothelial activity, nitric oxide also reacts with reactive oxygen species to increase the synthesis of potent cellular oxidants, which accumulate as oxidative stress (19). Notably, migraine patients also experience higher levels of asymmetric dimethylarginine—a nitric oxide synthase inhibitor that correlates to endothelial dysfunction and oxidative stress—which suggests that migraine headache results from inadequate prevention of oxidative damage (19). Compared to the neurological mechanisms of headache, the vascular influences of migraine might seem minimal, but when considering the crucial impact of neurovascular signaling on central processes, consistent cerebral vasodilation assists in the expansion of chronic headache pain through cutaneous allodynia and thus establishes the disabling severity of untreated headache disorders.

### 1.3. Sex differences in migraine

Migraine is more commonly diagnosed with a higher rate of disability in cisgender (i.e., individual's gender identity matches assigned sex at birth) women than men (4, 21). Although migraine rates between the two sexes are similar prior to puberty, post-pubertal women have a higher migraine prevalence and frequently report higher pain intensity scores, longer headache attack durations, increased risk for headache reoccurrence, increased migraine-related disability, and increased occurrence of cutaneous allodynia as compared to cisgender men (21, 22). Furthermore, puberty introduces a particular female-specific subtype known as menstrual migraine that occurs either before or during menstruation when estrogen levels decline; conversely, pregnancy is known to generally improve migraine attacks, except in the case of migraine with aura (21). These sex-mediated differences suggest that migraine symptomology is linked to ovarian hormone fluctuations, although the molecular mechanisms driving these hormonal fluctuations and how they impact migraine pathology remain to be fully elucidated.

**Figure 1:**
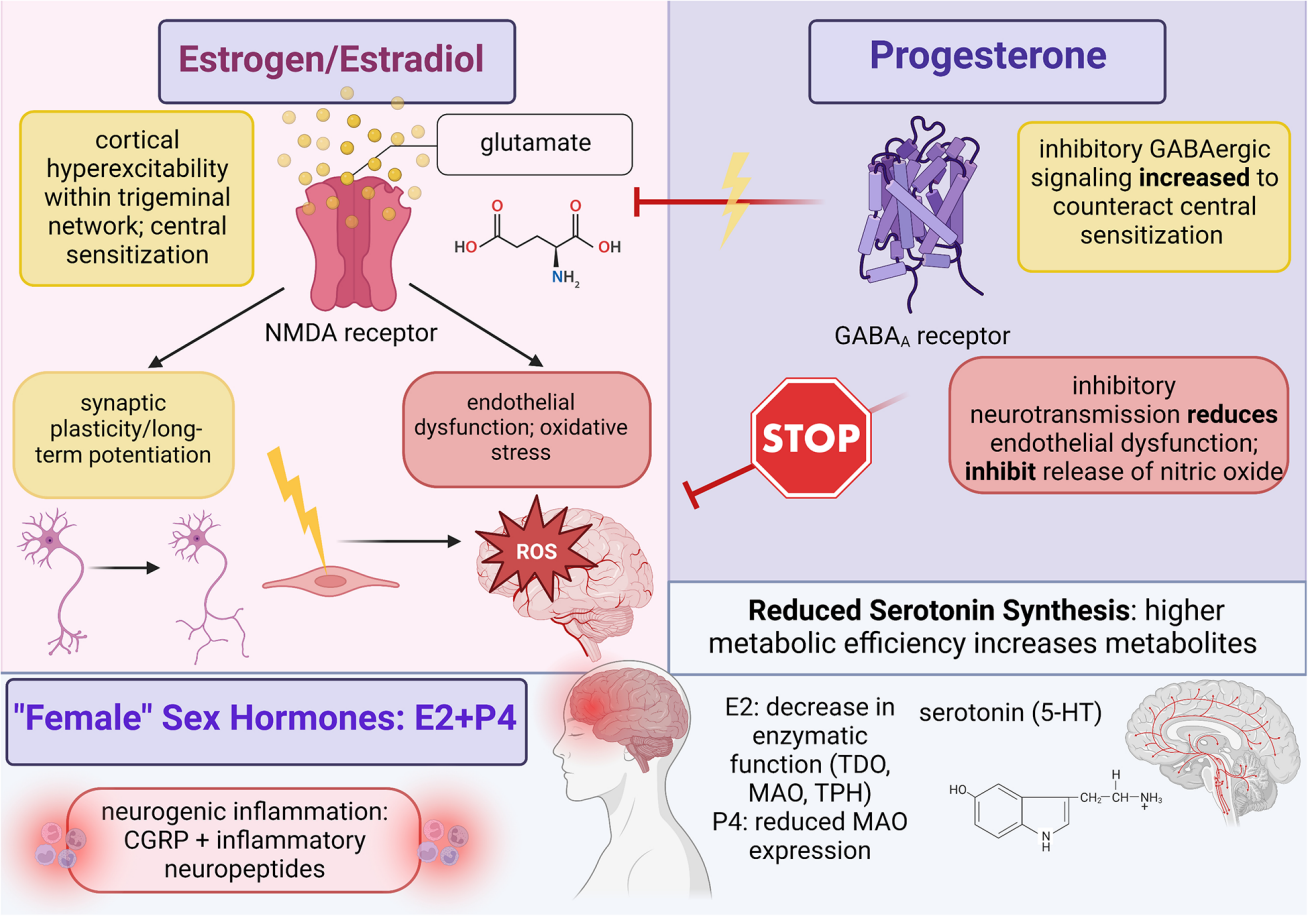
Summary of female sex hormone (estrogen and progesterone) involvement in migraine mechanisms; created with BioRender.com.

Research on sex differences usually refers to the hormonal differences between female sex hormones (e.g., estrogen, progesterone) and male sex hormones (e.g., testosterone), but the correlation between migraine and menstrual sex hormone fluctuations reveals that the physiological concentration as well as the type of sex hormone is critical for headache pain. The human menstrual cycle typically lasts 28 days and is divided into two stages: the follicular and luteal phase. In the follicular phase, progesterone levels remain low while estradiol steadily increases. Ovulation then triggers a sharp decline in estradiol simultaneous with rising progesterone levels; when progesterone peaks halfway through the luteal phase, estradiol remains slightly elevated until both hormones drop back down to baseline (23), Figure 1A. Comparatively, the estrus cycle of rodents only lasts four days and is divided into the diestrus, proestrus, estrus, and metestrus phases. Diestrus features declining progesterone and rising estradiol levels that peak during early proestrus only to be reversed by progesterone in late proestrus. Both estradiol and progesterone experience a sharp decline during the estrus phase before progesterone increases slightly in metestrus (23), Figure 1B. While many animal studies consistently only include male and ovariectomized female murine models to avoid ovarian hormone fluctuations, techniques for staging the estrus cycle in rodents—such as vaginal cytology, histological examination of reproductive organs, vaginal wall impedance, and urine biochemistry—have proven to be reliable and accurate measurements to determine gonadal hormone concentrations (24, 25). Notably, since the National Institute of Health (NIH) outlined a new policy in 2014 for preclinical studies to address sex as a biological variable (26), the number of publications discussing sex differences has topped 400 (PUBMED, keywords: female, rodent, migraine headache; search dates: 2014–2022). While this increase in reporting is a step forward, numerous studies still fail to stage the estrus cycle and account for gonadal hormonal fluctuations.

Sex hormones are lipid signaling messengers that can enact long-lasting effects on neurovascular structure and function. To provide more comprehensive treatment for cisgender women/men and to expand the much-needed area of research on chronic conditions within transgender/gender non-conforming (shortened to trans* as a gender-inclusive umbrella term) patients who receive gender-affirming sex hormone replacement therapy (HRT), a comprehensive analysis on the impact of hormonal fluctuations in migraine-related pathological processes and on nociceptive processing is required. This review will first examine pre-existing, chronic pain-related health disparities among trans* patients to determine current healthcare needs regarding pain management and migraine headache. The medical necessity of gender-affirming treatment and current barriers to healthcare access will then be integrated to emphasize that gender affirmation is critical to reestablish important health measures—such as patient self-efficacy and trust in the healthcare system—that will grant trans* patients with the necessary skills needed to improve pain outcomes. Due to the significant knowledge gap between gender-affirming care and physical health disparities, research on sex hormone activity in migraine pathology will be summarized through cisgender patient data as it pertains to ovarian HRT (estrogen/progesterone) and androgen HRT (testosterone). Further gaps in knowledge and future research directions will be identified as needed to improve healthcare practice and to continue gender-affirming healthcare for LGBTQ + patients.

## 2. Hormone mechanisms implicated in migraine

### 2.1. Incidence

When explicitly examining sex differences in primary headache, cisgender women report a higher migraine prevalence of 18% compared to 6% among men (21), whereas cisgender men have a higher incidence of cluster headache (27). Together, this prevalence further implicates distinct roles for hormones in headache pathophysiology. With respect to migraine in this current review, cisgender women also had an increased diagnosis rate for migraine with aura at 2.6%–10.8% while cisgender men had an incidence between 1.2% and 3.7% (28). Although migraine aura is typically associated with visual disturbances, the mechanism underlying aura, cortical spreading depression, has an anterior spread across the cortex and thus can affect multiple sensory systems (3). Therefore, sex differences determined that strictly visual aura occurs in 1.8% of men and 4.2% of women; sensorimotor aura occurs in 0.3% of men and 1.7% of women; and combined visual/sensorimotor aura occurs in 0.4% of men and 1.9% of women (28). Clinical evidence from cisgender women demonstrates a trend between acquired migraine and/or worsening headache pain during the second and third trimesters of pregnancy; specifically, elevated ovarian hormones during pregnancy tend to increase rates of migraine with aura (21). Murine models of migraine headache suggest that estrogen increases CSD susceptibility to induce pronociceptive states, but the mechanism of action has yet to be clarified (21). How CSD alters pain perception, at least in comparison to other migraine mechanisms, is also not fully known. From a Danish population study that conducted interviews with 4,000 patients, the incidence of migraine without aura was reported as two times higher than headache with aura (29). Therefore, migraine mainly depends on mechanisms such as central sensitization, oxidative stress, and defective serotonin synthesis to disrupt pain processing pathways.

### 2.2. Estrogens and migraine

Estrogen is categorized as a “female” sex hormone synthesized within the ovaries to induce female sexual maturity; however, this simplification fails to consider the role of estrogen in male physiology as well as on other biological systems in cisgender women. Estrogen incorporates three hormones that have different functions based on the stage of female reproduction: estradiol is the main hormone that is sustained over a woman's lifespan while estrone and estriol are weaker forms that become dominant during menopause and pregnancy, respectively (30). Estrogen hormones can bind to two receptors: estrogen receptor alpha (ERα) and estrogen receptor beta (ERβ). Research still needs to clarify the connection between estrogen receptor signaling and migraine mechanisms since both ERα and ERβ are expressed in the central nervous and cardiovascular systems, but current evidence proposes that ERβ could be responsible for initiating migraine headache since studies that targeted ERβ demonstrated more profound effects on the central nervous and immune systems than ERα (31). Since ovarian hormones can cross the blood-brain barrier, ERβ activity likely contributes towards neurogenic inflammation and central sensitization in migraine (31).

The high prevalence of disabling migraine features among cisgender women implies that fluctuating levels of estrogen are critical for migraine development, but assumptions based on female physiology and the effects of hormonal signaling currently restrain the understanding of migraine to a system primed for estrogen. The difference in prevalence between migraine with aura during high estrogen and migraine without aura during estrogen withdrawal suggests that in addition to promoting migraine mechanisms, higher levels of estrogen increase CSD susceptibility and encourage significant headache-related disability through neurovascular dysfunction. Headache studies conducted using animal models demonstrated that female mice have intrinsically lower CSD thresholds than male mice (32), but when female sex hormones were administered to murine neocortical slices, CSD frequency and amplitude increased (33). Although this evidence reflects clinical data among cisgender women and trans* patients on estradiol HRT, CSD involvement in nociceptive neurotransmission seems minimal compared to its own role in sensory disruption and to the widespread efficiency of central sensitization. Because migraine without aura carries a higher disease burden, examining the role of estrogen in cortical hyperexcitability, neurogenic inflammation, and persistent vasodilation is critical to improve headache pain outcomes for all patient populations.

Given that CSD could be an initiating mechanism of migraine in patients, increased CSD susceptibility from female hormones indicates that estrogen has a critical role in triggering migraine wherein higher hormonal concentrations correlate to worse pain outcomes. However, clinical data from both cisgender and trans* patients differentiate the effectiveness of estrogen on male and female physiology. The introduction of estradiol to male physiology enables rapid CSD and central sensitization induction since low levels of pre-existing estrogen are insufficient to dominate testosterone activity, but for patients with female physiology, the developmental role of estrogen for sexual reproduction could require hormones that exceed healthy estradiol concentrations to propagate migraine mechanisms. During Tanner stage IV which occurs after puberty onset, patients with female physiology report estradiol levels between 15 and 85 pg/ml while male patients report a maximum of 38 pg/ml. In adulthood, female patients experience fluctuating levels of estradiol within the range of 15–350 pg/ml that varies according to the menstrual cycle (34); during the follicular phase, estradiol levels decrease to 20–80 pg/ml before reaching maximum concentrations between 200 and 500 pg/ml during ovulation. By the midluteal phase, estradiol levels can vary from 60 pg/ml to 200 pg/ml (35). For adult males, estradiol increases slightly, but overall concentrations ideally remain within 10–40 pg/ml (34). Although trans* patients can customize the method of application (e.g., oral pill, subcutaneous/intramuscular injection, absorption through skin) and concentration of hormone received to achieve personal definitions of gender expression, the Endocrine Society guidelines for gender-affirming HRT state that full binary feminization is complete when serum estradiol levels fall between 100 and 200 pg/ml, which is obtained from effective doses ranging between 90 pg/ml to 200 pg/ml (36). Until further research can clarify the minimum hormonal concentration of estradiol needed to induce migraine within male and female physiology, the treatment of chronic headache in both cisgender and trans* patients will remain retrospective as treatment goals focus more on disease management rather than risk evaluation and avoidance. Thus, by summarizing the known interactions between estradiol signaling and migraine headache, data from cisgender patients can clarify expected outcomes and risks associated with headache and gender-affirming HRT.

Estradiol likely activates estrogen receptors in the central trigeminovascular system to facilitate the disruption of neurotransmitter and ion concentration gradients that augment the synaptic efficiency of pain-processing neurons (6). Further research needs to be conducted to clarify the G-protein signaling pathways between activated estrogen receptors and trigeminal neurons as current evidence only discusses the impact of estradiol signaling, but overall, estrogen-induced activity provokes migraine by increasing excitatory neurotransmission and decreasing inhibitory signaling through the neurotransmitter gamma-aminobutyric acid (GABA) (28). The significant sex disparity in migraine could indicate that either estrogen is required to originate CSD and central sensitization or that abnormalities in estradiol activity prime the dysfunctional neurovascular conditions through which migraine can manifest. Estrogen might be responsible for CSD induction because potassium and calcium ion channels coupled to estrogen receptors can increase extracellular concentrations of neuronal calcium; this imbalanced gradient will provoke a greater influx of calcium ions into trigeminal neurons which triggers synaptic depolarization and lowers the activation threshold for nociceptive neurotransmission (28). Central sensitization further maintains trigeminal cortical hyperexcitability through underlying neurological processes that foster neural synchronicity, mainly by upregulating the release of glutamate. Estrogen also augments the expression and activity of N-methyl-D-aspartate (NMDA) receptors on glutamatergic neurons; specifically, long-term potentiation in response to headache improves trigeminal synaptic efficiency by increasing the density of dendritic spines on NMDA-dominant neurons (37). However, the significant dysfunction of migraine from elevated hormones indicates that estradiol activates processes that not only progress synaptic plasticity and cortical excitability but also hinder recovery through inhibitory signaling. Elevated levels of estradiol reduce serum magnesium ions, and since magnesium ions are necessary to block NMDA channels from excessive activation, atypical estrogen activity disrupts the sensitive balance between excitatory and inhibitory signaling (38). Estrogen also decreases the tone of GABA-ergic neurons by uncoupling GABA_B_ receptors from the G-protein-coupled receptor attached to potassium ion channels; this suppression of neuronal hyperpolarization lowers activation thresholds because hyperpolarization is necessary to return neuronal activity back to baseline (38).

While only the outcomes from estrogen activity on migraine mechanisms have been reported, several theories have been put forth and examined. The kynurenine (KYN) pathway contains several metabolites vital to regulating excitatory and inhibitory signaling in the central nervous system. Kynurenic acid (KYNA) is a competitive antagonist that binds to the glycine site of the GluN1 subunit of NMDA receptors while other metabolites—such as quinolinic acid (QUINA) and xanthurenic acid (XA)—increase cortical excitability as orthosteric agonists that bind to the GluN2A-D subunit of NMDA receptors. QUINA and XA also activate type-2 metabotropic glutamate receptors (39). Migraine abnormalities were noted when Curto et al. (39) found that chronic migraineurs expressed reduced levels in KYN (−32%), KYNA (−25%), and QUINA (−80%) as well as elevated levels in XA (+28%). Chauvel et al. (37) explored the impact of these metabolites on CSD propagation and discovered that administration of the KYNA precursor l-kynurenine (L-KYN) combined with probenecid (PROB) to inhibit organic acid efflux reduced parieto-occipital CSD frequency more dramatically in female rats than in male rats; additionally, both sexes also demonstrated a significant reduction in CSD frequency at the frontal recording site. During estrus cycling, female rats who received L-KYN without PROB demonstrated a decrease in CSD frequency in both the frontal and parieto-occipital sites for every phase of the estrus cycle, although significance was only reached during diestrus. Further sex differences showed that female rats have a higher intrinsic capacity to synthesize KYNA because in response to L-KYN application, cortical KYNA increased by 400 times in male rats but only 100 times in female rats, suggesting that female rats have a higher baseline (37). Higher KYNA levels could protect against cortical hyperexcitability because KYNA also functions as a non-competitive inhibitor for α-7-nicotinic acetylcholine receptors (nAChR); when cortical KYNA is increased by less than twofold in murine models, glutamate release was downregulated by 50%. Female rats also demonstrated higher cerebral nAChR density compared to male rats; therefore, given its potency, KYNA could be a promising treatment for migraine that minimizes glutamate neurotoxicity and prevents further chronic pain from repeated trigeminal sensitization (37).

Central sensitization consists of two critical components: cortical hyperexcitability facilitated through increased glutamate expression and enhanced synaptic efficiency through long-term potentiation; therefore, revealing the contributions of estrogen signaling on both aspects will improve headache pain relief. Clinical studies on migraine sex differences reported that in addition to elevated CGRP, cisgender women also experience fluctuating CGRP expressions within the central and peripheral nervous systems that correspond to estradiol concentrations across the menstrual cycle (18). Evidence from animal models demonstrated that high CGRP and low expressions of CGRP-receptor encoding mRNA were found in the medulla tissue of female but not in male rats, which suggests that CGRP receptor synthesis and/or expression could be regulated by ovarian sex hormones (18). Furthermore, oophorectomized female rats revealed that elevated hormones increased CGRP expression in the dorsal ganglion while hormone withdrawal increased CGRP expression in the dorsolateral periaqueductal gray area, so further research should clarify estradiol concentrations with CGRP activity and pain-processing cortical regions (28). Preliminary theories can be drawn from Cetinkaya et al. (40) in which intact male, intact female, and oophorectomized female rats were treated with female sex hormones; however, because female rats were pre-treated with progesterone, conclusions from this study rely on underlying progesterone signaling. Although estrogen might increase CGRP concentrations under specific conditions, exogenous dosing of estradiol to both male rats and progesterone-primed female rats reduced serum CGRP. Since female rats were examined during proestrus which features elevated estradiol and minimal neuropeptide release, progesterone might modify the physiological environment of pain-processing networks to generate migraine headache during periods of estrogen withdrawal. Prior research indicates that migraine susceptibility increases in response to prostaglandins activated by low estradiol levels, so future research should determine the effects and signaling pathways in which progesterone and estrogen dually interact to promote migraine (40). Karkhaneh et al. (41) measured CGRP levels within blood nonnuclear cells following pharmacological and physiological doses of 17β-estradiol to compare migraine headache among cisgender women. Pharmacological doses increased CGRP mRNA among women with menstrual migraine and healthy female controls; conversely, physiological doses decreased CGRP plasma levels and mRNA expression among female migraineurs only (28). This effect is likely accomplished through the estrogen-induced downregulation of bradykinin B2 and interleukin-1β receptors which are thought to facilitate the release of CGRP from sensory neurons (41).

Estradiol signaling seems to have a minimal role in directly perpetuating cerebral vasodilation since a significant portion of endothelial dysfunction and oxidative stress is proportional to the sensitized strength of trigeminal neurotransmission. However, research on the vascular contributions to migraine with sex hormones is still needed to elucidate the full impact of estrogen. In a study that compared plasma concentrations of nitric oxide among cisgender women with either menstrual migraine, non-menstrual migraine, or non-headache controls, enhanced activation of the nitric oxide pathway during the luteal phase leads to increased nitric oxide synthesis that aligns with reports of oxidative stress markers peaking during the late follicular and early luteal phases (42). Estrogen also regulates vascular tone through nitric-oxide-mediated metabolic pathways that produce cerebral vasodilators, such as cyclic guanosine monophosphate (cGMP), cyclic adenosine monophosphate (cAMP), and prostacyclins (28). ERα receptors on endothelial cells increase nitric oxide synthase activity by stimulating the protein phosphatidylinositol 3-OH kinase, and as nitric oxide is released, soluble guanylate cyclase is activated to produce cGMP, which triggers neurogenic inflammation among cranial blood vessels (19).

Estrogen-mediated disruptions in essential biomolecules bolster the efficiency of trigeminal neurotransmission at the cost of regulatory processes that are necessary to prevent excessive cortical hyperexcitability and cerebral vasodilation; therefore, since migraineurs demonstrate higher levels of serotonin (5-HT) during the ictal phase of headache but lower levels during the interictal phase, estrogen could influence the synthesis of neurotransmitters like serotonin (43). Although serotonin has a total of seven functional receptors, only 5-HT1, 5-HT2, and 5-HT3 have been identified as contributors to migraine development since these receptors are found on trigeminal nerve endings and can thus influence trigeminal neurotransmission (44). 5-HT1 and 5-HT2 are both G-protein-coupled receptors whose activation triggers secondary messenger signaling cascades: 5-HT1 decreases cellular concentrations of cAMP to control long-term potentiation and regulate gene transcription for protein expression while 5-HT2 isoforms increase cellular inositol triphosphate (IP3) and diacylglycerol (DAG). 5-HT2 receptors have been implicated in control of the menstrual cycle, and since increased IP3 stimulates calcium release from the endoplasmic reticulum to mediate the activity of downstream targets involved in gene expression and synaptic strengthening, serotonin receptors could contribute towards central sensitization and neurogenic inflammation in migraine (45). In fact, several studies have shown that elevated estrogen during the menstrual cycle upregulates 5-HT levels while states of estrogen withdrawal reduce 5-HT to the diminished baseline observed in migraine patients (44). Furthermore, 5-HT3 receptors function as nonselective, ligand-gated Na+/K + ion channels that impact dopamine and acetylcholine release as well as influencing the GABAergic inhibitory system. Durham et al. (46) demonstrated that endogenous activation of 5-HT1 receptors triggered calcium signaling pathways that inhibited CGRP gene transcription by suppressing promoter activity through cAMP response element (CRE) and a cell-specific enhancer; specifically, increased calcium inhibits CRE binding protein (CREB) activity by inhibiting CREB phosphorylation and stimulating phosphatase activity. In the presence of a selective 5-HT agonist, sustained elevation of intracellular calcium prevents activation of the mitogen-activated protein kinase (MAPK) pathway which is responsible for CGRP expression (44).

In addition to generating intermediate metabolites that destabilize excitatory and inhibitory cortical signaling, the KYN pathway also reduces serum 5-HT levels in migraineurs by metabolizing the amino acid tryptophan into KYN metabolites at the expense of serotonin synthesis. Michael et al. (47) demonstrated potential sex differences in migraine corresponding to tryptophan metabolism when it was shown that after oral tryptophan administration, urinary excretion of L-KYN metabolites was significantly higher among cisgender women than men, suggesting that ovarian hormones increase tryptophan metabolism via the KYN pathway to promote migraine mechanisms like central sensitization (37). Tryptophan 2,3-dioxygenase (TDO) and indolamine 2,3-dioxygenase (IDO) are rate-limiting enzymes required for tryptophan metabolism along the KYN pathway, and several studies indicate that estrogen can induce TDO activity through the hypothalamic-pituitary-adrenal axis as well as upregulate IDO expression in antigen-presenting cells. For serotonin synthesis, the rate-limiting enzyme tryptophan hydroxylase (TPH) catalyzes tryptophan into 5-hydroxytryptophan (5-HTP) which is then decarboxylated by aromatic L-amino acid decarboxylase to produce serotonin. Serotonin metabolism is then catalyzed by the enzyme monoamine oxidase (MAO) to generate 5-hydroxyindoleactic acid (5-HIAA) (37). When applied to serotonin levels in migraine, low levels of 5-HT during the interictal phase positively correlated with high plasma concentrations of 5-HIAA; this aligns with the theory that migraine headache results from enhanced metabolic flux and increased enzymatic efficiency (43). Aggarwal et al. (48) supported this by showing that ovariectomy of female rats reduced TPH gene expression by 30% and increased MAO gene expression by 4-fold compared to intact female rats; however, following treatment with estradiol, TPH and MAO gene expression returned to baseline. In response to TPH and MAO enzymatic activity, ovariectomy generated a 50% and 30% decrease in gene expression for 5-HT1A and 5-HT1B receptors, respectively. Additionally, ovariectomy also reduced 5-HT2A receptor gene expression by 80%. Estradiol treatment abolished this effect with a 150% increase in 5-HT1A mRNA, a 20% increase in 5-HT2A mRNA, and complete restoration of baseline mRNA expression for 5-HT1B receptors (48). Cummings (49) postulated that since serotonin proliferates vasoconstriction on trigeminovascular nerve endings and blood vessels, 5-HT serves a neuroprotective role against migraine headache. However, low 5-HT levels from estrogen-mediated disruptions are insufficient to hinder excessive cerebral vasodilation, and evidence suggests that serotonin is upregulated during headache as an attempt to mitigate trigeminal pain (44). Serotonin signaling appears critical in modulating disproportionate levels of neurogenic inflammation since 5-HT agonists normalized CGRP levels in migraineurs (44). Along with rate-limiting enzymes and 5-HT levels, Aggarwal et al. (48) also noted that ovariectomy increased CGRP expression by 600% that was reduced to 150% following estradiol treatment. Further analysis is needed to connect serum 5-HT levels in migraineurs with 5-HT receptor activity and stimulated neurovascular dysfunction.

An overview of the current understanding of estrogen signaling on neurovascular migraine mechanisms is summarized in Figure 1 to address potential migraine susceptibility.

### 2.3. Progesterone hormone contributions

Although estrogen hormones are incredibly efficient at accumulating dysfunction within the trigeminovascular system, the role of fluctuating progestin hormones with estradiol signaling could clarify gaps in understanding between migraine headache, hormonal states, and pain symptomology. Progesterone has two receptors: progesterone receptor alpha (PRα) and progesterone receptor beta (PRβ). Although murine models demonstrate higher expressions of one receptor isoform over the other, human cells contain equivalent amounts of PRα and PRβ, which suggests that the PRα-PRβ heterodimer is the dominant molecular species for humans (50). Therefore, progesterone activity relies on the expression of progesterone-receptor coregulators to control hormone responsiveness (50). Reciprocal interactions between estrogen and progesterone could be integral in either prevention or maintenance of migraine because estrogen and progesterone receptors often colocalize wherein estradiol receptor activity could potentially influence progesterone receptor expression (28). Yet, the diversity and complexity of coregulators and their signaling pathways have limited research on central processes of progesterone signaling. By analyzing the impact of endogenous progesterone on migraine, current gaps on the role of estrogens in migraine and interactions between physiological sex hormones can be clarified.

### 2.4 Combined estrogen and progesterone effects

Despite limited evidence on progesterone-mediated processes, the impact of progesterone on CSD and central sensitization can be inferred through studies that analyze the combined effects of estradiol and progesterone signaling on trigeminal neurotransmission; observed differences between combined female sex hormones and estrogen are typically attributed to progesterone. Thus, progesterone seems to regulate estrogen-mediated activity as a neuroprotective agent that can prevent cortical hyperexcitability. When ovarian hormones were applied to the trigeminal nucleus caudalis of female rats, estrogen induced hyperresponsiveness while progesterone triggered hyporesponsiveness (28). Progesterone and its metabolite allopregnanolone mitigate central sensitization by upregulating GABA_A_ receptor activity within neurons of the trigeminal nucleus caudalis (51). Additionally, GABA_A_ receptors exhibit significant synaptic plasticity during the menstrual cycle; several studies reported synaptic plasticity within cortical regions implicated either in trigeminal pain-processing or in the descending control network of nociceptive neurotransmission, which includes the hippocampus, thalamus, and periaqueductal gray area (52). Since progesterone peaks during the luteal phase of menstruation, estrogen-mediated hyperexcitability could be offset with progesterone as a therapeutic target that increases the synaptic efficiency for inhibitory signaling among trigeminal neurons. However, the association between menstrual migraine and states of estradiol withdrawal likely suggests that serum levels in female migraine patients are insufficient to fully reverse migraine development. Progesterone signaling could be supplemented endogenously by increasing the synthetic pathway of 3a-hydroxy-5a-pregnan-20-one within glial cells since this metabolite is predominantly active at GABAergic receptors (53). The caveat to this potential therapeutic role depends on the strength of trigeminal neurotransmission between estrogen and progesterone activity. Given that central sensitization is reinforced through long-term potentiation and synaptic plasticity, inhibitory signaling upregulated by progesterone and metabolites may not be enough to adequately hinder migraine. Morphological changes to synaptic connections within the trigeminal nucleus caudalis following estradiol treatment were not immediately eradicated during estrogen withdrawal, indicating that cortical hyperexcitability persists even in the presence of progesterone and heightened inhibitory signaling (28). Furthermore, progesterone application to neocortical slices mimicked estrogen activity by increasing CSD frequency and amplitude, suggesting that progesterone might bolster migraine headache in the absence of estrogen to trigger menstrual migraine (37). Future research should clarify the mechanistic action of progesterone in ovariectomized and menstrual female headache models to determine how female sex hormones interact to either support or eliminate chronic headache pain.

Preliminary research exploring both progesterone and estrogen signaling on migraine can be summarized according to the following theory: although progesterone can theoretically protect against migraine development by counteracting estrogen-induced cortical hyperexcitability with inhibitory signaling, endogenous progesterone among migraine patients is insufficient to reverse synaptic plasticity along sensitized pain-processing pathways. This discrepancy likely explains menstrual migraine during periods of estrogen withdrawal since estradiol signaling might be necessary to initiate CSD and central sensitization. Thus, because estrogen and progesterone reach their maximum concentration during the luteal phase of the menstrual cycle, progesterone might disrupt neurovascular pain signaling such that migraine invoked from estradiol is sustained by progesterone (54). When Cetinkaya et al. (40) investigated the impact of ovarian hormones on CGRP levels, ovariectomized female rats pretreated with progesterone had reduced CGRP in response to estradiol treatment. Since female rats were evaluated during the proestrus phase of estrus cycling, high levels of estrogen combined with lower amounts of neuropeptides suggests that even without estrogen priming, progesterone can trigger a mild neurogenic inflammatory response that generates headache without central sensitization. Progesterone also increased CGRP release from trigeminal ganglion neurons in male rat tissue. Thus, Cetinkaya et al. (40) proposed that a “threshold of serum progesterone levels exists above which migraine is provoked and below which it is prevented” (55). By repeating the experimental conditions in intact female rats, Cetinkaya et al. (40) showed that endogenous estrogen and progesterone produces a similar upsurge in plasma CGRP that aligned with the proposed progesterone-mediated increase in neuropeptide expression. Therefore, elevated concentrations of estradiol might not be a prerequisite for migraine headache; instead, female sex hormones might generate inherent vulnerabilities within trigeminal neurovascular pathways that increase migraine susceptibility.

Although further research is needed to determine whether progesterone could serve as a therapeutic agent to reduce trigeminal central sensitization, Jayaraman and Pike (56) revealed that progesterone's functional role as either a positive or negative mediator for persisting migraine mechanisms depended upon sustained serum presence. When ovariectomized female rats were exposed to progesterone over the course of several weeks and/or months, prolonged progesterone downregulated mRNA expressions of ERα and ERβ which suppressed the release of neurotrophins involved in synaptic long-term potentiation (i.e., BDNF, nerve-growth factor, and neurotrophin 3). Furthermore, the minimum threshold defining this effect was determined at fifteen hours wherein pretreatment with prolonged progesterone completely inhibited estrogen-mediated neuroprotection (56). Bearing these conditions in mind, previously conflicting evidence regarding the role of progesterone on migraine pathology can be re-examined to clarify gaps in understanding; however, future research is necessary to discern the contributing neurovascular pathways activated by progesterone. In response to migraine propagated by estradiol signaling, progesterone seems to eradicate central sensitization and oxidative stress through enhanced inhibitory GABAergic neurotransmission and blocked release of nitric oxide for vasoconstriction (54). Under certain conditions, however, progesterone can reinforce headache pain through estrogen-mediated disruptions to the serotonergic system of migraineurs that generates less neuroprotection against cortical hyperexcitability. Receptor colocalization between estrogen and progesterone on serotonergic neurons within the raphe nuclei provides a connection between hormone signaling and alterations in 5-HT expression; thus, progesterone assists estradiol in increasing the metabolic rate of 5-HT synthesis by modifying key rate-limiting enzymes. When spayed monkeys were administered either estradiol, progesterone, or combined hormones, both estrogen and progesterone were able to reduce 5-HT synthesis through reduced MAO_A_ gene expression in the dorsal raphe and several hypothalamic nuclei (54). D’Andrea et al. (57) studied serum serotonin levels in response to fluctuating hormones across the menstrual cycle and determined the phase in which maximum and minimum platelet serotonin levels were reached. High sex hormone concentrations induced maximum 5-HT levels during the late follicular and early luteal phases while low sex hormones initiated minimum 5-HT levels during the early follicular and late luteal phases (55). Similar evidence was reported by Fioroni et al. (58) in which patients with menstrual migraine had greater MAO_B_ activity and higher 5-HIAA levels but lower serotonin concentrations during the late luteal phase compared to the follicular phase (55). Because progesterone demonstrates such versatile adaptability in response to estradiol signaling, studies that focus on delineating the progesterone threshold are thereby needed to form a complete understanding of progesterone and migraine susceptibility. One possible direction to explore centers on cyproterone acetate, which is a progestin hormone with anti-androgen activity to suppress testosterone synthesis via the hypothalamic-gonadal axis, that could be utilized as a potential countermeasure against migraine-related neurovascular activity by reducing 5-HIAA levels to promote 5-HT synthesis (52).

### 2.5. Testosterone hormone therapy on migraine

Current research on the role of testosterone signaling within the central neurovascular network is limited to expected pain outcomes, thus necessitating future studies. This gap in understanding is especially important considering sex hormones can diffuse across the blood-brain barrier and local testosterone synthesis has been observed along the central nervous and cerebrovascular systems (59). Testosterone is a synthetic metabolite for estrogen biosynthesis, so androgen levels among female migraineurs could be further reduced by physiological mechanisms that are selective for estrogen (60). Migraine among cisgender men likely relies on this defective pathway to explain the appearance of migraine headache despite the higher disparity of chronic pain among cisgender women. Furthermore, because estrogen levels are so critical in promoting central sensitization, upregulated estrogen synthesis at the cost of reduced testosterone in male physiology could be sufficient to trigger and sustain migraine. However, minimum physiological hormone levels have not yet been determined for migraine susceptibility, so the conditions under which testosterone neuroprotection could be effective are unknown. This is further complicated by the lack of standardized testosterone levels for cisgender men and woman across different ages: measurements of testosterone are often unreliable and imprecise due to significant variabilities on how to accurately measure testosterone, especially since androgen concentrations remain relatively stable compared to the frequent fluctuations of ovarian hormones (61). Since androgen receptors (AR) within central neurovascular pathways can also colocalize in the same areas as estrogen and progesterone receptors, gaps in knowledge on the central neurovascular activity of traditional sex hormones neglects the complex and intricate signaling of hormonal messengers and headache-related disability (62).

The androgen deficiency model of migraine proposes that sufficient concentrations of serum testosterone are needed to sustain a level of neuroprotection against estrogen-mediated processes; however, despite the advantage of testosterone in patients with female physiology, increasing testosterone levels in cisgender men and trans* patients with male physiology in the hope of offsetting estradiol signaling might not provide the same benefit. While neurogenic inflammation is promoted by estrogen and inflammatory neuropeptides like CGRP, testosterone targets the caspase-6-mediated microglial signaling pathway to control neuropathic pain exclusively in male physiology. Sorge et al. (63) determined that spinal TLR4 (Toll-like receptor 4) receptors are responsible for regulating microglial inflammation such that activation generated hyperalgesia and allodynia exclusively in male mice, even though neurogenic inflammation triggers long-term potentiation and central sensitization in both sexes (4). When the microglial inhibitor minocycline was applied to castrated males and testosterone-treated females, only female mice experienced a reduction in allodynia, implying that neurogenic inflammation in males requires testosterone. Indeed, mechanical allodynia was reduced in intact male mice through administration of several microglial inhibitory factors such as minocycline, microglial toxin, purinoceptor 4 receptor antagonist, and deletion of the BDNF-encoding gene (64). When compared to neurogenic inflammation through CGRP, the caspase-6 signaling pathway is initiated when some nerve injury releases caspase-6 from axonal terminals in the spinal cord; microglial cells are then activated via tumor necrosis factor (TNFα) receptors type I and type II to elicit central sensitization. Microglia also release p38 MAPK to further incite long-term potentiation, and this form of synaptic plasticity seems essential for central sensitization development in male physiology since spinal administration of a p38 inhibitor effectively reduced neuropathic pain in male mice. Chronic pain states in cisgender men might depend on this pathway more so than CGRP inflammation because Kronschlager et al. (65) determined that this gliogenic form of long-term potentiation can independently spread throughout various nociceptive pathways via microglial activity and the diffusion of signaling messengers across cerebrospinal fluid (64).

Within the scope of literature that examines androgen receptor activity on central trigeminal neurotransmission, clinical proposals on the impact of testosterone within migraine pathology have mainly investigated the association between androgens and observations in headache pain relief. Until more is known about androgen signaling, testosterone has been suspected to directly reverse the estrogen-mediated cortical vulnerability to CSD propagation and central sensitization through similar molecular pathways as progesterone. Rose and Braidman (66) noted that testosterone successfully inhibited the estrogen-induced upregulation of TDO expression through the hypothalamic-pituitary-adrenal axis. Although the exact cellular mechanism remains unclear, a potential explanation could lie in the testosterone metabolites 5α-androstane-3α, 17β-diol (3α-diol) and 5α-androstane-3β, 17β-diol (3β-diol) which have been shown to directly regulate estrogen receptor activity and hinder cortical hyperexcitability through GABAergic signaling. Therefore, CSD propagation could be curtailed either through 3α-diol activity as an allosteric modulator of GABA_A_ receptors or through 3β-diol activity as a ligand for estrogen receptors (60). Eikermann-Haerter et al. (67) explored CSD suppression in response to androgen hormones within the migraine subtype known as familial hemiplegic migraine to elucidate the effectiveness of testosterone vs. its metabolites. A missense mutation in the CACNA1A gene, which is responsible for encoding the pore-forming α1A-subunit of voltage-gated calcium ion channels, causes calcium channels to open during more negative membrane potentials and to delay channel inactivation. Because these calcium channels remain active for longer periods, increased calcium ion influx within presynaptic neurons lowers trigeminal activation thresholds and primes the central nervous system for repetitive and persistent headache episodes. When murine headache models were treated with testosterone, chronic exposure was more efficient than acute at reversing CSD susceptibility, and since progesterone levels in cisgender women could be too low to completely abolish trigeminal synaptic plasticity, introduction of testosterone to female physiology seems to function as the antithesis to estrogen by effectively replacing trigeminal cortical hyperexcitability with greater analgesic efficiency from the descending modulatory pain system. This finding is further supported when testosterone was administered alongside the AR antagonist flutamide to confirm the involvement of testosterone-dependent pathways (67). If migraine patients indeed suffer from reduced serum testosterone due to deficits within androgen and estrogen biosynthetic pathways, testosterone metabolites could be utilized to bolster central neuroprotection and reduce headache pain severity by offsetting testosterone metabolism. The non-aromatizable androgen hormone 5-α-dihydrotestosterone (5αDHT) shows some promise as an androgen metabolite because it can regulate excessive NMDA activation through inhibiting irreversible neuronal depolarization and cellular apoptosis at high NMDA concentrations with larger NMDA-induced currents (67).

Although endogenous testosterone is presumed to be too low of a concentration to support neuroprotective processes in female physiology, compensatory analgesic mechanisms generated by female physiology could represent inadequate attempts to eliminate the damaging side effects of migraine. Thus, the hormonal profile of female physiology could encourage pronociceptive states, and therefore explain, in part, the binary sex difference of headache pain between cisgender men and women. When combined with the major effect of testosterone and estrogen on pain, migraine in cisgender men seems to be caused by disruptions in sex hormone synthesis wherein estrogen hormones are upregulated at the expense of testosterone. Shields et al. (68) and Romiti et al. (69) published studies that reported a positive correlation between low serum testosterone and migraine in cisgender male patients that also aligned with data gathered in other primary headache disorders, suggesting that the imbalance between high female and low male sex hormones is a critical component in primary headache disorders. This theory is supported by van Oosterhout et al. (70) wherein concentration ratios between testosterone and estrogen were analyzed in cisgender men with migraine. During the interictal phase, male migraineurs demonstrated lower ratios due to upregulated estrogen despite retention of normal testosterone levels; conversely, serum testosterone increased during the ictal phase, which could potentially represent an attempt to retroactively regulate estrogen's involvement in pain promotion (70). Furthermore, the effectiveness of testosterone as a potential therapeutic treatment was explored in several clinical studies wherein more migraine patients benefited from the combined sex hormone treatment of estradiol and testosterone than patients who received estradiol only (71).

The efficacy of testosterone in reducing headache pain implies that testosterone could participate in the same processes as progesterone to reverse estrogen-mediated pronociception; Since both ovarian hormones lower the availability of serum 5-HT in migraine patients, testosterone could bolster the efficacy of serotonin binding by regulating 5-HT receptor expression through the colocalization of AR with 5-HT2 receptors. Castration of male rats reduced 5-HT2AR binding site density in the nucleus accumbens and the frontal, cingulate, and piriform cortices (72). Following treatment with either testosterone propionate or estrogen benzoate, 5-HT2AR binding site density in these regions was reestablished back to or greater than pre-castrated male rats; additionally, testosterone and estrogen also increased mRNA expression for 5-HT2AR receptors and 5-HT transporters in the dorsal raphe nucleus. Since elimination of female sex hormones by ovariectomy in female rats demonstrated a similar pattern of reduction and restoration of 5-HT2AR binding sites, these functional similarities between male and female hormones reflect the theory in which the conversion of testosterone to estrogen can promote migraine pain states. Given that the androgen metabolite 5αDHT demonstrated no effect, migraine pain outcomes for patients with male physiology seem to depend on testosterone activity and the level of estradiol to regulate trigeminal neurotransmission (72). Further research that outlines the impact of sex hormone synthesis on various neurovascular migraine mechanisms is needed to estimate the antinociceptive qualities of testosterone, especially since the vascular pathways of testosterone have yet to be fully explored and defined. Although vasoconstriction can reduce the intensity of headache pain, testosterone activates ARs and induces a vasodilatory response that is theorized to stabilize cerebral blood flow and inhibit oxidative stress, but evidence proving this theory is still being debated (62, 73, 74).

Despite testosterone's traditional categorization as a “male” sex hormone produced within the testes and adrenal glands for male sexual maturity, androgens may not inherently function in direct opposition to female sex hormones as implied (62). Clinical evidence from trans* headache studies in which gender-affirming testosterone HRT improved pain outcomes supports the theory that anti-inflammatory properties from testosterone can improve endogenous pain relief for migraineurs with female physiology (73, 75). Given that estradiol HRT can overcome testosterone-induced neuroprotection to disrupt trigeminal signaling, hormonal contributions to migraine need to explore the involvement of testosterone within both male and female physiology to clarify the antinociceptive potential of androgens.

**Figure 2:**
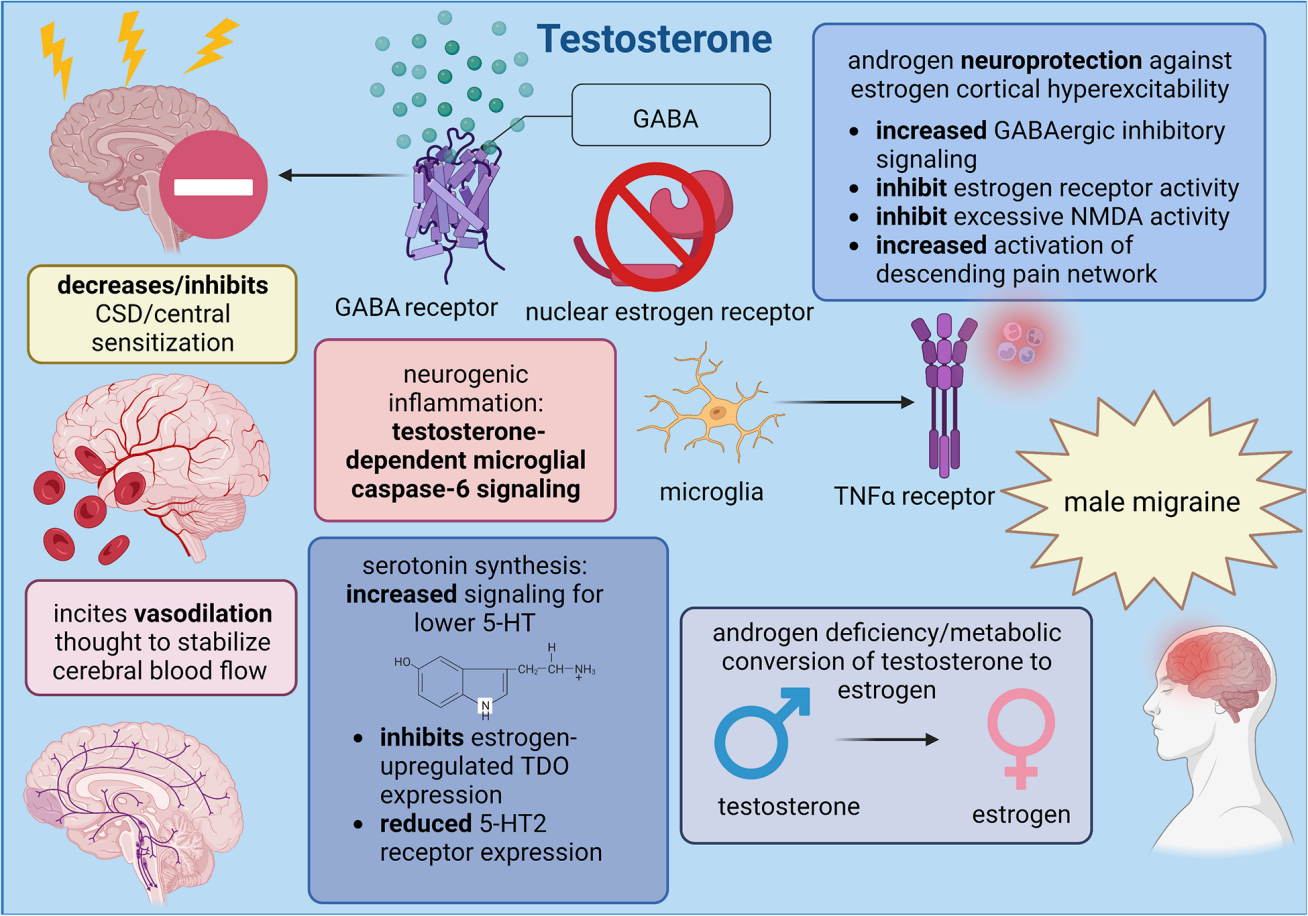
Summary of male sex hormone (testosterone) involvement in migraine mechanisms; created with BioRender.com.

Considering the significant lack of research defining testosterone signaling on central neurovascular functions, the role of testosterone in migraine is currently restrained to its exogenous role as analgesic agent within female patients. Notably, evidence of endogenous testosterone in male physiology implies that androgen metabolism into estrogen and microglial-mediated neurogenic inflammation generates migraine headache in male patients. Standardized levels of testosterone should first be determined to improve the accuracy of testosterone and estrogen concentrations so that pain management for cisgender men might be improved, but the inclusion of trans* individuals could clarify the developmental relationship between testosterone in male physiology and estrogen in female physiology with the propagation of nociceptive migraine mechanisms. A summary of the endogenous and exogenous effects of testosterone on migraine are provided in Figure 2.

### 2.6. Other sex hormones and migraine

Much of the language used to discuss sex differences relies on the binary perception of biological sex, and research investigating the role of sexual dimorphism in different pathological conditions often remains confined to the major gonadal hormones: estrogen, progesterone, and testosterone. Table 1 summarizes the involvement of these hormones on crucial migraine mechanisms. However, given the widespread function of sex hormones in facilitating signaling cascades outside of their respective sex-defined reproductive systems, clinical research should also evaluate the potential signaling effects of minor sex hormones such as prolactin, follicle-stimulating hormone, luteinizing hormone, and gonadotropin-releasing hormone.

**Table 1:** Overview of male/female sex hormone signaling on critical migraine mechanisms.

Migraine mechanisms	Estrogen/Estradiol *female sex hormone*	Progesterone *female sex hormone*	Testosterone male sex hormone *potential metabolic transformation into estrogen
CSD/central sensitization	• Increased activation of NMDA receptors + increase in glutamate release • Long-term potentiation via synaptic strengthening • Cortical hyperexcitability	• Increased inhibitory signaling via GABA neurons • Long-term exposure: reduced BDNF + neurotrophins • Reduces cortical hyperexcitability	• Decrease in cortical hyperexcitability ○ 3α-diol increases GABAergic inhibitory signaling ○ 3β-diol inhibits estrogen receptor activity ○ 5αDHT inhibits excessive NMDA activity • Increased activation of pain-descending network
Neurogenic inflammation	• Proinflammatory neuropeptides ○ Upregulated CGRP	• Proinflammatory neuropeptides ○ Upregulated CGRP	• Capase-6 inflammatory signaling
Oxidative stress	• Increased endothelial activation • Increased vasodilation via NO signaling	• Downregulated release of NO • Increased vasoconstriction	• Reduced oxidative stress from stabilizing cerebral blood flow
Serotonin synthesis	• Reduced serotonin synthesis ○ Decreased activity of rate-limiting enzymes (TDO, MAO, TPH)	• Reduced serotonin synthesis ○ Decreased MAO expression	• Increased serotonin synthesis ○ Inhibition of estrogen-induced upregulation of TDO • Reduced 5-HT receptor expression

Overview of sex hormone signaling on migraine mechanisms.

#### 2.6.1. Role of prolactin in migraine pain

Prolactin is predominantly synthesized from lactotroph cells expressing G-protein-coupled dopamine D2 receptors in the anterior pituitary but can also be produced from the gonads, endothelial cells, and adipose tissue to regulate endocrine, immune, and nervous system activity (76, 77). Serum prolactin levels are regulated by a short feedback loop in which synthesis of the neurotransmitter dopamine within tuberoinfundibular dopamine (TIDA) neurons in the arcuate nucleus (ARC) is promoted by high prolactin levels to inhibit prolactin signaling (76). Much like estrogen, upregulated prolactin might contribute to migraine via sensitization of trigeminal nociceptive neurons (78). A correlation between high prolactin and migraine was recently described in which administration of the dopamine D2 receptor antagonist cabergoline proved effective in reducing intense, unilateral face pain involved in prolactinoma-associated headache (76). Furthermore, despite male and female mice demonstrating similar density in TIDA nerve terminals, sex differences revealed that TIDA activity is intrinsically higher in females while low testosterone and orchidectomy enhanced TIDA activation in males (79). Gender-affirming hormone therapy also facilitated a 25% decrease and 193% increase in prolactin levels among transgender men and women, respectively (80).

The prolactin receptor (PRL) gene on chromosome 5 encodes three different isoforms: long (PRL-L), intermediate, and short (PRL-S), but only PRL-L and PRL-S will be considered since unlike humans and rats, mice lack intermediate PRL receptors (77). PRL-L and PRL-S are type I cytokine receptors expressed within trigeminal ganglion neurons and dural afferent fibers that transmit signals through the Janus tyrosine kinase 2/signal transducer and activator pathway. PRL-L activates signaling pathways involved in long-term potentiation—such as the Src family of tyrosine kinases, phosphatidylinositol 3-kinase (PI3K)/protein kinase B, MAPK, and serine/threonine kinase Nek3-Vav2-Rac1 pathways—which implies that prolactin could reinforce central sensitization and neurogenic inflammation. However, animal studies suggest that trigeminal hyperexcitability is incited through PRL-S activation and TRP channel sensitization—specifically, potentiation of TRPV1 in the trigeminal ganglion and the TRP subfamilies V1, A1, and M8 in dorsal root ganglia from PI3K and protein kinase C*δ* downstream signaling—which generates central sensitization that is more prominent in female murine models (77).

Prolactin-induced sensitization imitates estradiol neurogenic inflammation by proliferating CGRP release from sensory neurons via the excessive activation of sensitized TRP channels. Although sensory neurons lack PRL, application of TRP channel activators to cultured female sensory neurons pretreated with prolactin resulted in amplified trigeminal neurotransmission due to enhanced calcium ion influx. Within male physiology, prolactin sensitization might require abnormally high concentrations to further support estrogen-mediated sensitization since males have lower PRL-L and PRL-S expressions in the dorsal root ganglion (78). PRL-S signaling could be necessary to prime neurogenic inflammation in migraine since isolated sensory neurons taken from both male and female PRL-knockout mice successfully reversed prolactin-induced TRPV1 sensitization upon PRL-S reintroduction. Furthermore, when PRL-S and PRL-L expressions were reestablished to a 1:1 ratio in PRL-knockout models, prolactin sensitization was completely abolished, implying that PRL-L expression is responsible for PRL-S inhibition (78).

When prolactin is considered alongside androgen and ovarian hormones, pronociceptive headache states fueled by estrogen can recruit prolactin-induced sensitization to further advance chronic pain since estrogen increases PRL receptor expression. Conversely, testosterone suppresses PRL expression to mitigate central sensitization, but since the molecular pathway defining this androgen-mediated phenomenon has yet to be explored, only coinciding interactions between estrogen and prolactin will be discussed. High concentrations of endogenous estrogen within female migraineurs correlate to an increase in PRL receptor mRNA in dorsal root ganglion neurons; however, because equal expressions of PRL-S and PRL-L were retained in both sexes regardless of estradiol concentrations, estrogen upregulation of PRL expression occurs independently from receptor isoform translation (78). Application of the translation inhibitor 4EGI-1 blocked prolactin-induced sensitization to male sensory neurons and mechanical allodynia in estrus-staged female mice by downregulating PRL expression, suggesting PRL-L receptors could be a therapeutic target for migraine. Additionally, knockdown of PRL-L resulted in persisting nociceptive hypersensitivity perpetuated by spinal NMDA activity and enhanced glutamate neurotransmission which affirms the proposed role of PRL-L in abolishing prolactin-induced migraine mechanisms (78).

**Figure 3:**
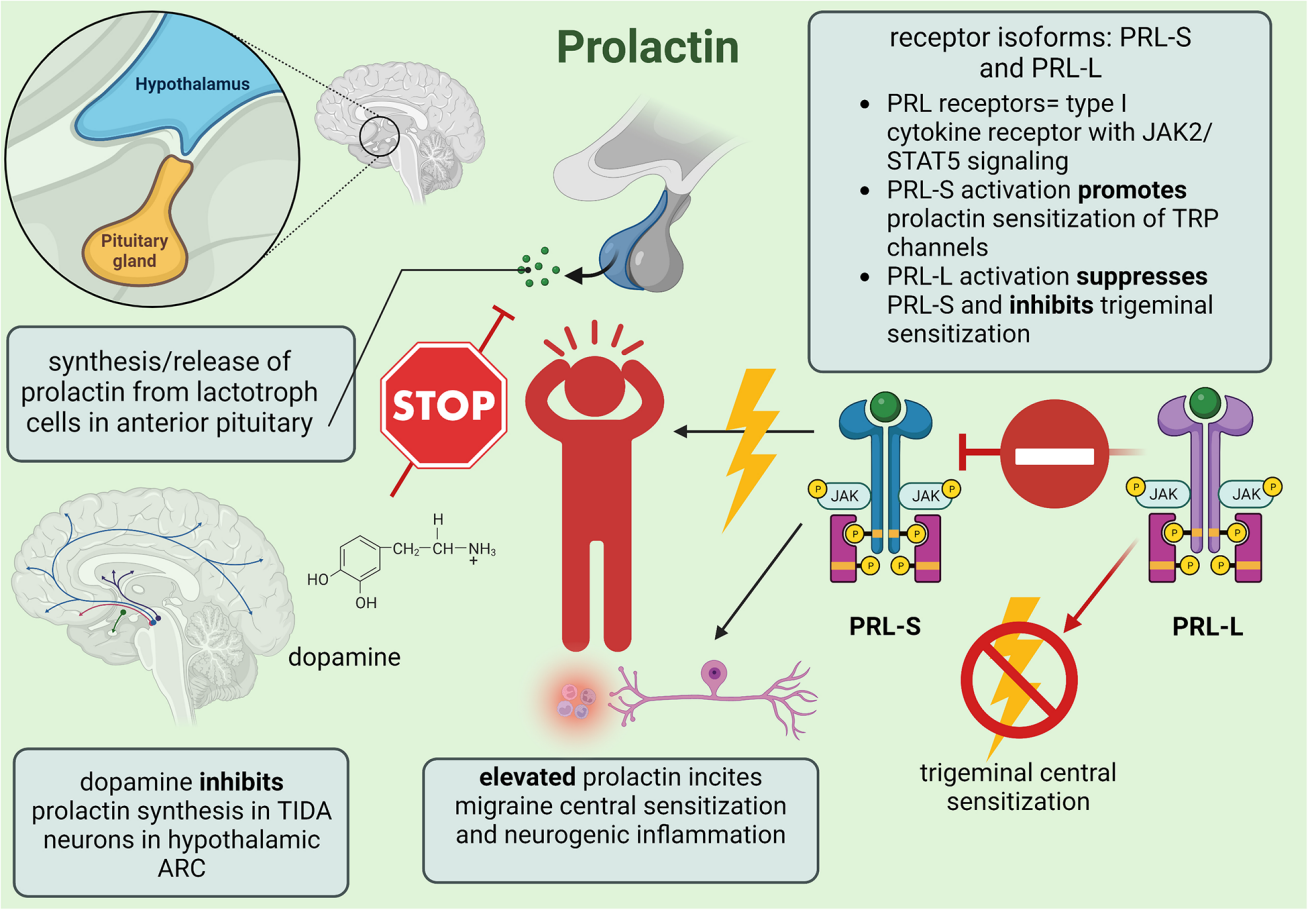
Summary of minor female sex hormone prolactin involvement in migraine mechanisms; created with BioRender.com.

Stress is a significant, albeit complex, migraine trigger that might involve prolactin-induced sensitization of the hypothalamic-pituitary-adrenal (HPA) axis and reduced efficacy of the kappa-opioid receptor (KOR) system to alleviate trigeminal nociception. Previous clinical studies reported that stress activates the hypothalamus of migraineurs to increase cortisol levels, resulting in a positive correlation between stress-induced priming and chronic headache frequency. Since stress increases prolactin and the endogenous KOR agonist dynorphin, central sensitization could decrease the analgesic efficiency of the KOR signaling pathway such that chronic stress would hinder endogenous recovery from headache (76). Watanabe et al. (76) investigated the impact of restraint stress (RS) and prolactin-induced sensitization using KOR^CRE^ mice to deduce sex differences in migraine. Prior to experimentation, murine models expressed KOR^CRE^ neurons in the dorsomedial/ventromedial hypothalamic nuclei and ARC as well as within tyrosine-hydroxylase-positive cells that were quantified as 80% for female and 70% for male mice. In response to RS, enhanced serum prolactin produced allodynia from latent sensitization in both male and female mice, although females experienced an intrinsically greater increase. When the KOR antagonist nor-binaltorphimine dihydrochloride was applied to the right ARC, serum prolactin was reduced likely due to the synchronized TIDA network (76). Combined with evidence that knockout of KOR expression effectively blocks RS-primed transient cutaneous allodynia and latent sensitization, hypothalamic KOR signaling seems to be a necessary component in prolactin-induced sensitization wherein repeated activation of KOR neurons in the ARC lowers female sensory activation thresholds. Furthermore, administration of clozapine-N-oxide—a G-protein-coupled DREADD (designer receptor exclusively activated by designer drug) specific agonist—significantly increased prolactin in KOR^CRE^/G_i_-DREADD female mice after a single dose while systemic application downregulated PRL-L expression in female trigeminal ganglion V1 division tissue, suggesting that upregulated prolactin might induce sensitization through the subsequent activation/deactivation of PRL-S and PRL-L, respectively. Repeated treatment with cabergoline prevented RS-primed allodynia among female mice only, and since this effect persisted when RS-primed females were treated with umbellulone to trigger CGRP release via TRPA1 channel stimulation, cabergoline represses prolactin release through TIDA inhibition (76, 81). Furthermore, since CGRP antagonists blocked umbellulone-induced allodynia completely in females but only partially in males, neurogenic inflammation within females includes prolactin-induced TRP channel sensitization for upregulated CGRP while males with intrinsically lower prolactin levels seem to rely on inflammatory caspase-6 signaling to promote migraine (76). However, the disinhibition of pituitary lactotrophs due to TIDA inhibition from endogenous opioids could increase male prolactin since acute inflammation facilitates prolactin release from extra-pituitary sources such as local lymphocytes and vascular endothelial cells (77). Therefore, pituitary prolactin in females and extra-pituitary prolactin in males likely causes neurogenic inflammation from prolactin-induced stimulation of immune system lymphocytes, macrophages, and neutrophils to proliferate proinflammatory and nociceptive cytokines (77). The mechanisms of prolactin's involvement in migraine headache are summarized in Figure 3.

#### 2.6.2. Luteinizing hormone/follicle-stimulating hormone

Luteinizing (LH) and follicle-stimulating (FSH) hormones are synthesized from gonadotropin cells in the anterior pituitary to amplify estrogen synthesis during the menstrual cycle (82). Several studies (83–86) reported no significant differences in plasma concentrations between female migraineurs and healthy controls which suggests a lack of involvement in propagating migraine mechanisms. Nevertheless, Li et al. (87) noted a positive correlation between LH/FSH levels and headache course in both cisgender men and women with primary headache disorders; however, this correlation likely reflects the role of elevated estrogen levels and dysfunction of the hypothalamic-pituitary-adrenal axis in increasing migraine susceptibility through the mechanisms discussed above.

#### 2.6.3. Gonadotropin-releasing hormone

Although the majority of gender-affirming healthcare focuses on medical interventions for trans* adults, trans* youth who wish to postpone the puberty of their assigned sex might receive puberty blockers—which are gonadotropin-releasing hormone (GnRH) agonists such as goserelin, leuprolide, and histrelin—to delay the development of secondary sex characteristics and prevent gender dysphoria—which is defined in the DSM-5 as the “psychological distress that results from an incongruence between one's sex assigned at birth and one's gender identity” (88, 89). Sex differences in migraine illustrate that up to one year before puberty, cisgender men and women share an incidence rate of 4%, so by delaying sex hormones, sexual dimorphism between male and female physiology could be halted to potentially mitigate the risk of migraine headache (21). The Endocrine Society Clinical Practice Guidelines currently state that puberty suppression can be induced beginning in pubertal Tanner stage 2. For patients assigned female at birth, GnRH agonists could hinder migraine development since hypoestrogenic states would enable menstrual suppression and thus would stabilize female sex hormone fluctuations (90).

GnRH is synthesized within hypothalamic neurons to regulate LH and FSH concentrations throughout the menstrual cycle; the activation of GnRH G-protein-coupled type I receptors on gonadotrophin cells in the anterior pituitary generates GnRH pulse frequencies wherein low pulses stimulate FSH and high pulses promote LH. Although female migraineurs demonstrated normal FSH/LH levels, dysfunction of the hypothalamic-pituitary-adrenal axis could cause abnormal GnRH pulse frequencies. Research demonstrated that persistent GnRH stimulation produces elevated LH:FSH ratios—amplifying the ovarian synthesis of androgen hormones—while minimal stimulation and abnormal GnRH serum levels were associated with hypothalamic amenorrhea (91). Despite the potential for increased androgen-mediated neuroprotection and repressed menstrual-related hormonal fluctuations to reduce migraine susceptibility, the imbalance of hormonal signaling might still induce headache via elevated estrogen and prolactin. Since intrinsic dysfunction of the HPA axis in both male and female physiology disrupts sex hormonal signaling, further research is needed to elucidate the influence of puberty blockers and sex hormone HRT on chronic pain, especially since trans* youth also face significant barriers regarding access to this form of gender-affirming healthcare.

## 3. Gender affirming hormone therapy and migraine in transgender populations

Despite the growing number of trans* patients, healthcare practices have yet to move beyond the perspective that this patient population only engages with the healthcare system to receive gender-affirming medical treatment. Research examining pre-existing health disparities among LGBTQ + patients has been recognized as a crucial gap in knowledge (92); however, in-depth analysis on specific conditions is lacking. Even fewer studies have discussed the possible interactions between gender-affirming medical treatments (i.e., HRT), physical disability, and overall health outcomes. This failure to adequately recognize the impact of gender diversity has serious consequences not only for trans* patients but also for some cisgender patients with nontraditional gender expressions or hormonal imbalances. Assumptions about the visibility of nonconventional gender identities combined with patient hesitation to disclose or discuss aspects of gender-affirming healthcare can cause confusion about healthcare needs and generate a sense of mistrust. Thus, research on sex differences cannot rely on the binary sex model to reliably predict trans* health outcomes. The significant sex disparity in chronic pain between male and female patients combined with gender biases about the experience of pain already negatively impacts health outcomes; sexism in healthcare settings can emphasize the dismissal of chronic pain within women who appear “emotional” or “sensitive” while men might minimize their symptoms, potentially resulting in fewer available resources for pain management (93).

While the exact number of trans* individuals with migraine is unknown, there are an estimated 1.4 million transgender adults (determined from data analysis of the 2016 Behavioral Risk Factor Surveillance System) and 39 million individuals with migraine in the United States (94). Based on the coupled mindsets of gender-informed healthcare and analysis of physiological hormone expressions, this analysis of migraine headache pathology with gender-affirming HRT can hopefully serve as an important guide to expand the consideration of physical health disparities for trans* patients as well as address the unmet need for pain management among LGBTQ + individuals. When investigating the incidence of migraine in trans* patients, a Dutch study conducted by Pringsheim and Gooren (95) found that transgender women who had completed sex reassignment surgery, used anti-androgens to suppress male sex characteristics, and received estrogen HRT to induce female sex characteristics had a rate of 26% for migraine diagnosis. Compared to cisgender women and men in the same population, gender-affirming HRT increased the rate of migraine from 7.5% among men to match the 25% incidence found among cisgender women. Furthermore, 54% of transgender women experienced migraine with aura which suggests that estradiol HRT facilitates CSD and other pro-nociceptive mechanisms (21, 95). A similar study conducted by Aloisi et al. (75) wherein chronic pain was evaluated found that three trans* patients acquired headache in response to estradiol HRT while an additional two had continuing symptoms from a pre-existing pain disorder. Transgender women on estradiol HRT reported alterations in pain perception that match the features of chronic headache among cisgender women: increased frequency, lasting for longer durations, and enhanced pain sensitivity/intensity. Additionally, although headaches were not delineated into primary headache diagnoses, transgender women described headache symptoms that reflect migraine specificity (i.e., significant headache-related disability and environmental triggers like light and sound) while headache patients who experienced worsening pain might reflect the transition from episodic to chronic migraine (75).

Although this risk of headache could be detrimental for the physical and mental wellbeing of an already vulnerable population, transgender men and gender non-conforming individuals who receive androgen HRT might experience some relief from migraine. Yalinay Dikmen et al. (96) investigated the incidence of primary headache disorders among transgender men who were receiving or had previously received testosterone HRT. They found that 36.4% of transgender men had been diagnosed with migraine and were 50% more likely to use gender-affirming HRT compared to 36.1% of patients with tension-type headache; however, individuals without a primary headache diagnosis had the highest rate of current HRT usage at an incidence of 80% (96). Transgender men with migraine more commonly reported menstruation as a headache trigger, supporting the notion that migraine initiated from fluctuations in ovarian hormones might be stabilized as testosterone concentrations eventually surpass estrogen levels—especially since testosterone therapy can prevent menstruation (97). As such, when testosterone HRT was examined for headache improvement, 50% of migraineurs reported a decrease in headache frequency compared to only 21.4% of patients with tension-type headache (96). Aloisi et al. (75) found that diagnosis of a headache disorder prior to HRT was more common in transgender men. Following testosterone therapy, six patients found that the quality of their pain improved while three patients found no change, and one patient reported an increase in headache severity. For those who experienced improvement, both the frequency and duration of headache episodes were significantly reduced. However, transgender men who did not experience headache prior to hormone treatment but acquired a headache disorder following use of testosterone indicated that headache pain established a moderate level of disability with pain features that worsened over time (75).

From the available evidence, estrogen HRT is associated with an increased risk for acquired migraine in transgender women while testosterone HRT could potentially provide some pain relief for transgender men with a pre-existing primary headache disorder. Although data from cisgender migraineurs has supported the role of estrogen in promoting migraine mechanisms, gaps in knowledge remain on the minimum physiological concentration of estrogen required to propagate chronic pain states; given that migraine has been observed during states of estrogen withdrawal (i.e., menstruation) and elevation (i.e., pregnancy), determining physiological hormone concentrations can clarify the association of sex hormones and migraine symptoms. Consenting trans* patients represent the ideal clinical population to investigate this theory as the natural diversity in gender identity and use of gender-affirming hormones allows researchers to clarify the issue of sex hormone functionality without subjecting cisgender patients to potential unwanted side effects. These individuals are also ideal in furthering progress for LGBTQ + health outcomes beyond mental health disorders and sexual diseases because current debates regarding the legality of gender-affirming care already establish the immediate need for improved LGBTQ + healthcare and better understanding on the long-term effects of HRT. Treatment of headache for cisgender patients can also be improved through trans* data by reframing the consideration of health with gender identity; notably, by replacing binary sex as a health factor with measures of serum sex hormone levels, mechanisms involved in chronic pain pathology can improve the accuracy of disease diagnosis and treatment as well as support pain outcomes hindered by sexist gender stereotypes.

## 4. Conclusion

Despite limited data in trans* individuals with migraine, cisgender data suggests that femininizing estradiol HRT increases the risk for acquired headache and chronic pain while masculinizing testosterone HRT is associated with improved pain relief for patients with a pre-existing headache disorder. Future studies are needed to directly evaluate the risk between gender-affirming HRT and migraine. Ovarian hormones like estrogen and progesterone promote migraine headache via CSD and central sensitization within the trigeminal nociceptive network through reactive species, CGRP, and an imbalance of excitatory/inhibitory neurotransmission while androgens like testosterone attempt to reduce excessive cortical hyperexcitability by bolstering neuroprotective and analgesic qualities. When the effects of sex hormones in cisgender and trans* patients are examined together, rapid sex hormone fluctuations create vulnerabilities through which heightened estrogen induces pronociceptive states and reduced testosterone fails to adequately establish trigeminal neuroprotection. Since many trans* individuals begin HRT when patients are already well into adulthood, pubertal hormones likely establish migraine susceptibility that can be aggravated further by estrogen HRT, thus putting transgender women at the most risk. Susceptibility could be curtailed through pubertal suppression and treatment with puberty blockers, but the same limitations currently impacting adult gender-affirming care also affect trans* youth, maybe even more so due to debates on parental rights to oversee the healthcare decisions of their children. Given the changing landscape of trans* patients seeking gender-affirming healthcare, more investigation into sex hormones on migraine pathology is warranted to determine the impact of gender-affirming HRT and potential chronic pain risk.

Research on LGBTQ + healthcare has largely focused on educating medical providers about the appropriate terminology to improve interpersonal interactions between patients and providers (98, 99). However, difficulties in receiving gender-affirming care and a lack of education on LGBTQ + healthcare needs can aggravate chronic conditions that necessitate recurrent engagement between patients and medical providers; complications in navigating the healthcare system increase the likelihood for healthcare avoidance and the use of harmful practices to self-manage symptoms (100). Gordon et al. (101) conducted a survey on gender non-conforming patients that determined nonconventional gender identities increased the risk for poor health outcomes; particularly, trans* patients had a significant risk for chronic pain and mobility impairments. A similar study examining the incidence of chronic health conditions among LGBTQ + patients determined that chronic pain, neurological disorders, and severe mental health issues (e.g., substance use disorder, depression) were overexpressed in trans* patients compared to cisgender populations (102). Minority stress theory argues that negative health outcomes among marginalized groups are perpetuated through the compounded stress of social stigma, discrimination, and violence; thus, health disparities within the LGBTQ + community are maintained by insufficient protective health measures, prejudice within healthcare settings, and access barriers to essential healthcare services (100). By reframing pre-existing physical health disparities, disease pathology and susceptibility within the context of sex hormones instead of binary sex categories chronic pain outcomes may be improved for all including LGBTQ+, gender non-conforming patients, and those conforming to traditional gender roles.
