# Supplementary figures and images for: Molecular mechanisms of hormones implicated in migraine and the translational implication for transgender patients
Source: Front Pain Res (Lausanne). 2023 Sep 19;4:1117842. doi: 10.3389/fpain.2023.1117842 (PMC10546064; doi:10.3389/fpain.2023.1117842)

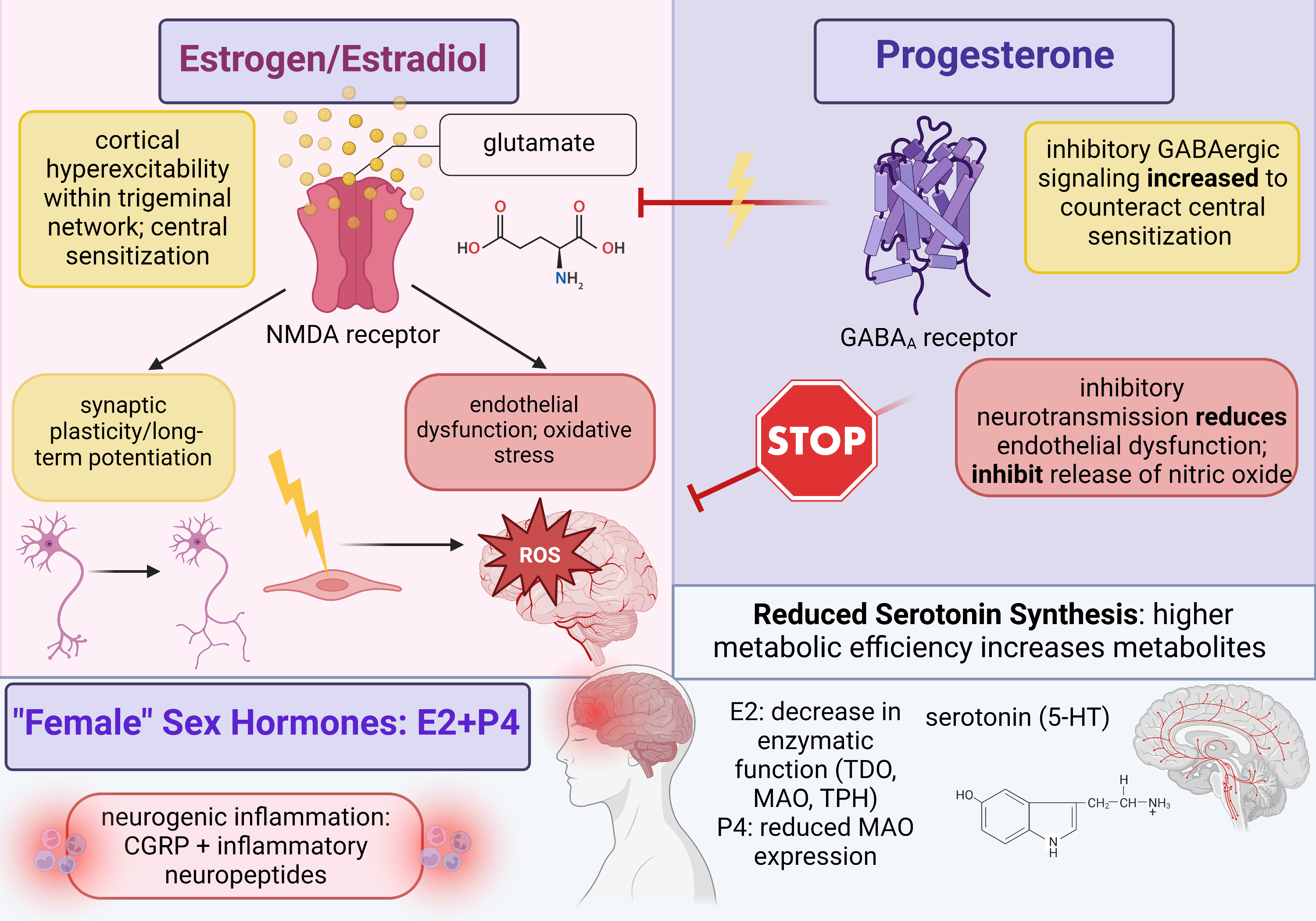

Supplement: Supplementary file 1 [file Datasheet1.zip › Data Sheet 1_v1/Supplementary Material Presentation/Female Sex Hormones_ Estrogen and Progesterone.jpg]

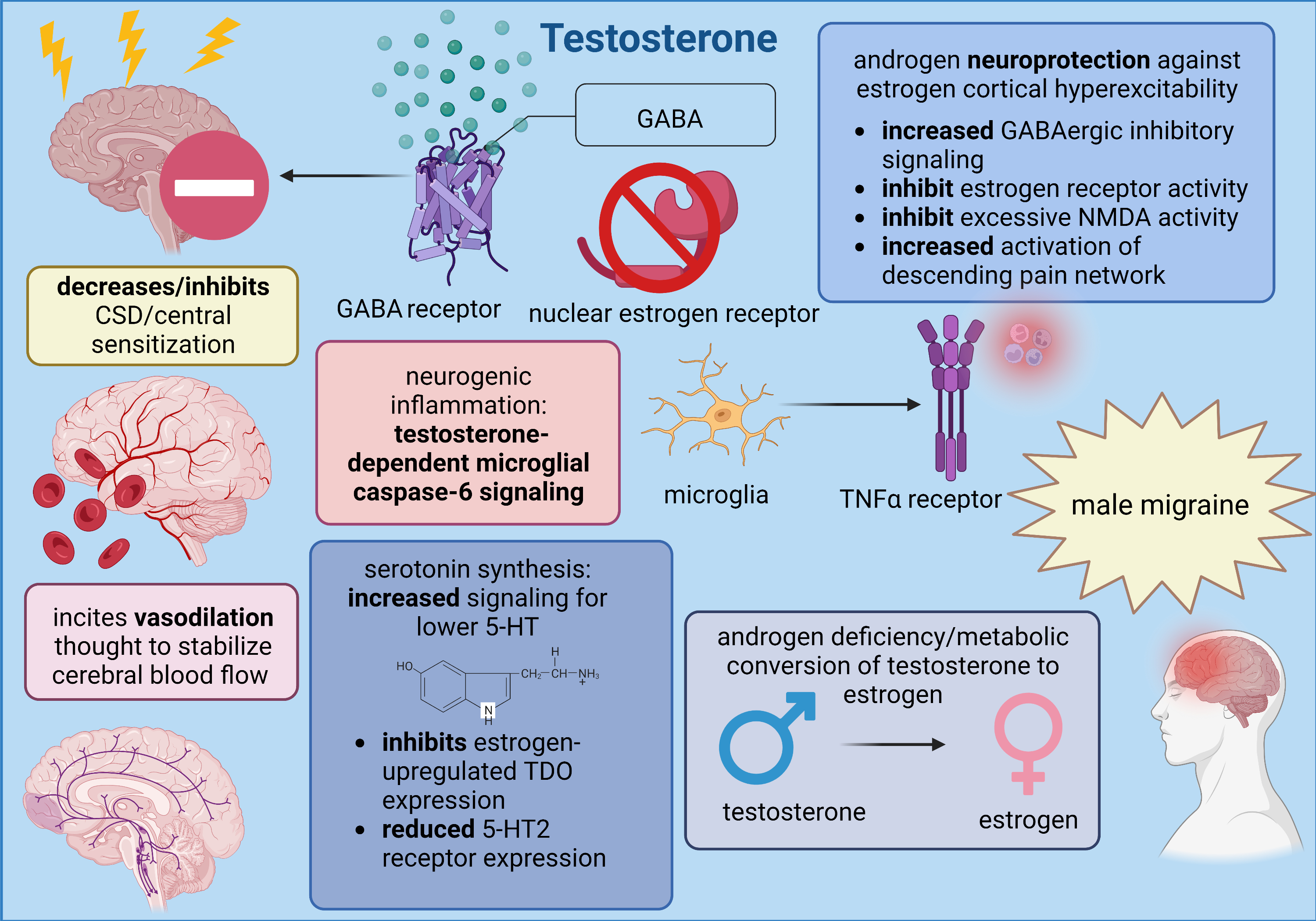

Supplement: Supplementary file 1 [file Datasheet1.zip › Data Sheet 1_v1/Supplementary Material Presentation/Male Sex Hormones_ Testosterone.jpg]

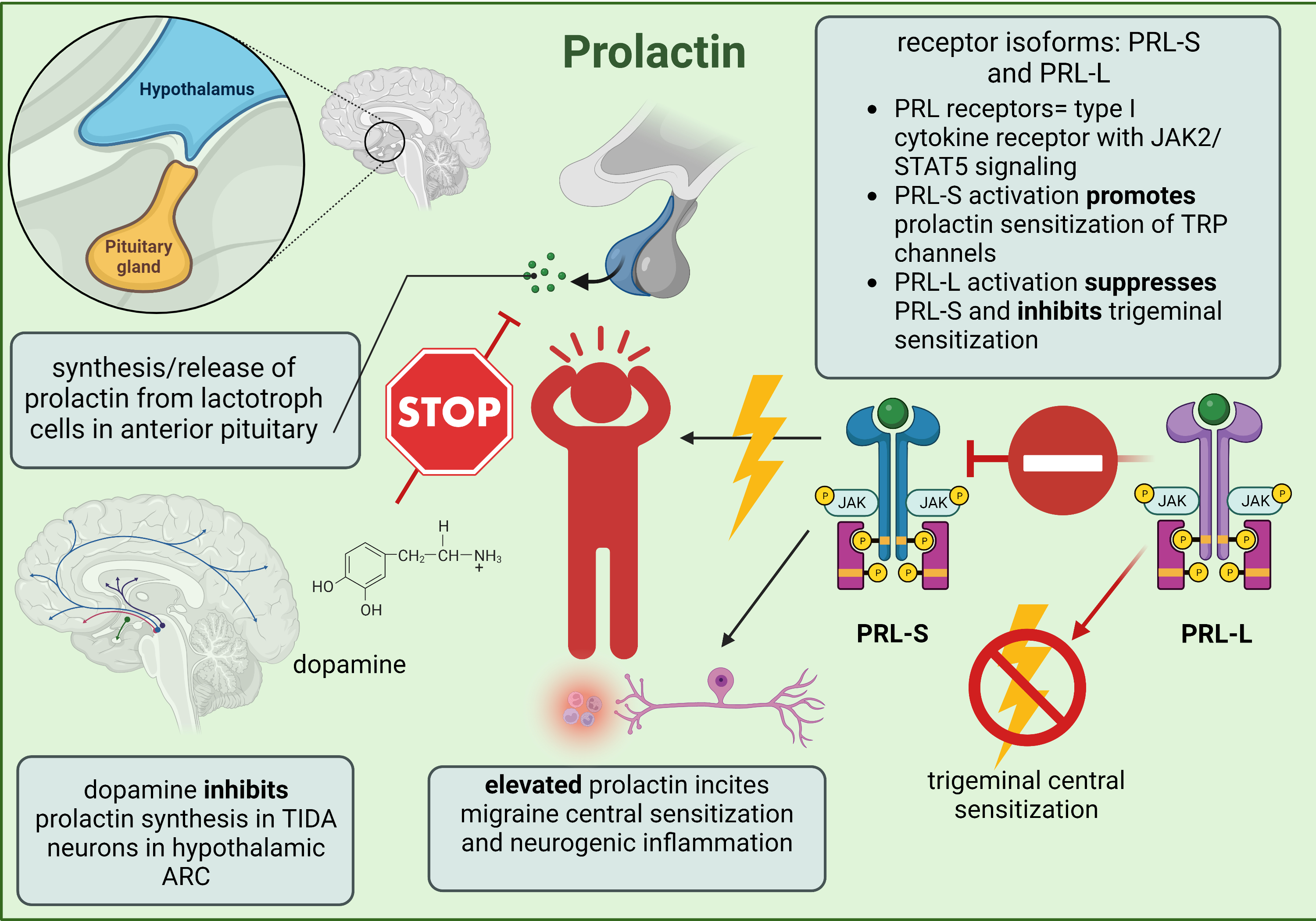

Supplement: Supplementary file 1 [file Datasheet1.zip › Data Sheet 1_v1/Supplementary Material Presentation/Minor Sex Hormones_ Prolactin.jpg]
